# Chromatography affinity resin with photosynthetically-sourced protein A ligand

**DOI:** 10.1038/s41598-024-59266-2

**Published:** 2024-04-15

**Authors:** Nisha A. Owens, Pieter H. Anborgh, Igor Kolotilin

**Affiliations:** 1 The School of Applied Science and Technology, Fanshawe College, London, ON Canada; 2 Solar Grants Biotechnology Inc., London, ON Canada

**Keywords:** Biotechnology, Plant biotechnology, Molecular engineering in plants

## Abstract

Green, photosynthesizing plants can be proficiently used as cost-effective, single-use, fully biodegradable bioreactors for environmentally-friendly production of a variety of valuable recombinant proteins. Being near-infinitely scalable and most energy-efficient in generating biomass, plants represent profoundly valid alternatives to conventionally used stationary fermenters. To validate this, we produced a plastome-engineered tobacco bioreactor line expressing a recombinant variant of the protein A from *Staphylococcus aureus*, an affinity ligand widely useful in antibody purification processes, reaching accumulation levels up to ~ 250 mg per 1 kg of fresh leaf biomass. Chromatography resin manufactured from photosynthetically-sourced recombinant protein A ligand conjugated to agarose beads demonstrated the innate pH-driven ability to bind and elute IgG-type antibodies and allowed one-step efficient purification of functional monoclonal antibodies from the supernatants of the producing hybridomas. The results of this study emphasize the versatility of plant-based recombinant protein production and illustrate its vast potential in reducing the cost of diverse biotechnological applications, particularly the downstream processing and purification of monoclonal antibodies.

## Introduction

The steady growth of the demand for therapeutic monoclonal antibodies (mAbs) necessitates the efficient, sustainable and affordable supply of the components required in the process of antibody purification, which accounts for more than two thirds of the overall downstream processing costs^1,2^. *Staphylococcus* (*S*.) *aureus* protein A (SpA) is the “golden standard” ligand used in the chromatography-assisted capture stage of the industrial antibody purification process^3–5^. The pH-sensitive, reversible molecular interaction of SpA with the Fc portion of most type-gamma immunoglobulins (IgGs) grants this ligand its supreme performance in chromatography purification applications, allowing removal of the majority of contaminating proteins in one step with excellent recovery yields^6–8^. Currently , the production of recombinant SpA for use as an affinity ligand primarily involves fermentation of *E. coli* and *Pichia pastoris* cultures^4,6,10^. The use of stationary bioreactors for the generation of the biomass that accumulates the desirable recombinant protein, i.e. “upstream production”, is associated with high operational and maintenance expenditures and essentially has a very limited ability for process scale-up from the initially designed production volumes^11,12^.

Stretching through millennia, human sustenance relied on the knowledge acquired and applied in agricultural practices and farming. Today’s plant biotechnology platform offers an attractive route for humanity to fulfill its “molecular farming” needs—the advantageous production of various recombinant proteins in order to address the ever-growing demand^13,14^. Plant-based synthesis and manufacturing of biologics constitutes, in fact, the “greenest” production process imaginable, where the biomass that accumulates the desired recombinant protein grows with utilization of abundant raw materials, such as CO_2_ from air, water and sunlight, while adding molecular oxygen to the atmosphere. The biomass produced is completely biodegradable, thus, its post-extraction disposal is ecologically friendly. Additional inherent beneficial features, such as the safety from mammalian pathogens and linear, easily affordable scalability further strengthen the position of plant-based production platforms as a valid alternative to conventional fermenters^11,15,16^. In particular, genetic engineering of the chloroplast genome (plastome) allows prolific expression of valuable recombinant proteins in stably-transformed plants’ chloroplasts (for reviews, see^17,18^). In addition to the high yields of the recombinant products, transplastomic plants as bioreactors offer advantages over nuclear-transformed plants in transgene containment due to maternal inheritance of the engineered traits and the lack of transgene transmission through pollen^19^. Additional beneficial features of plastome engineering include the absence of unwanted and detrimental “positional effects” leading to transgene silencing, pertinent to nuclear transformants, as well as the possibility to engineer several traits in one transformation step due to the ability of plastids to transcribe and translate operons^20^. In search of valuable and useful protein candidates for photosynthesis-driven mass-production, we successfully engineered tobacco chloroplasts to express a recombinant variant of SpA, producing a transplastomic line, a “green plant bioreactor”, with yields reaching ~ 250 mg of the recombinant protein per 1 kg fresh leaf biomass. The recombinant SpA was extracted, purified and covalently coupled to agarose beads, thus formulating a chromatography affinity resin (PlantA resin) that was used to efficiently purify monoclonal antibodies (mAbs) from the supernatants of the mAb-producing hybridomas. The purified mAbs were proven functional when used as a primary antibody in Western blots for immunodetection of their dedicated protein antigens. Our results demonstrate the absolute relevance of plant-based production for cost-effective, eco-friendly, and large-scale manufacturing of the recombinant SpA ligand for use in chromatography-assisted purification of mAbs to fulfill the growing needs of humanity for affordable antibody-related therapeutics.

## Results

### SpA bioreactor line engineering

**Figure 1:**
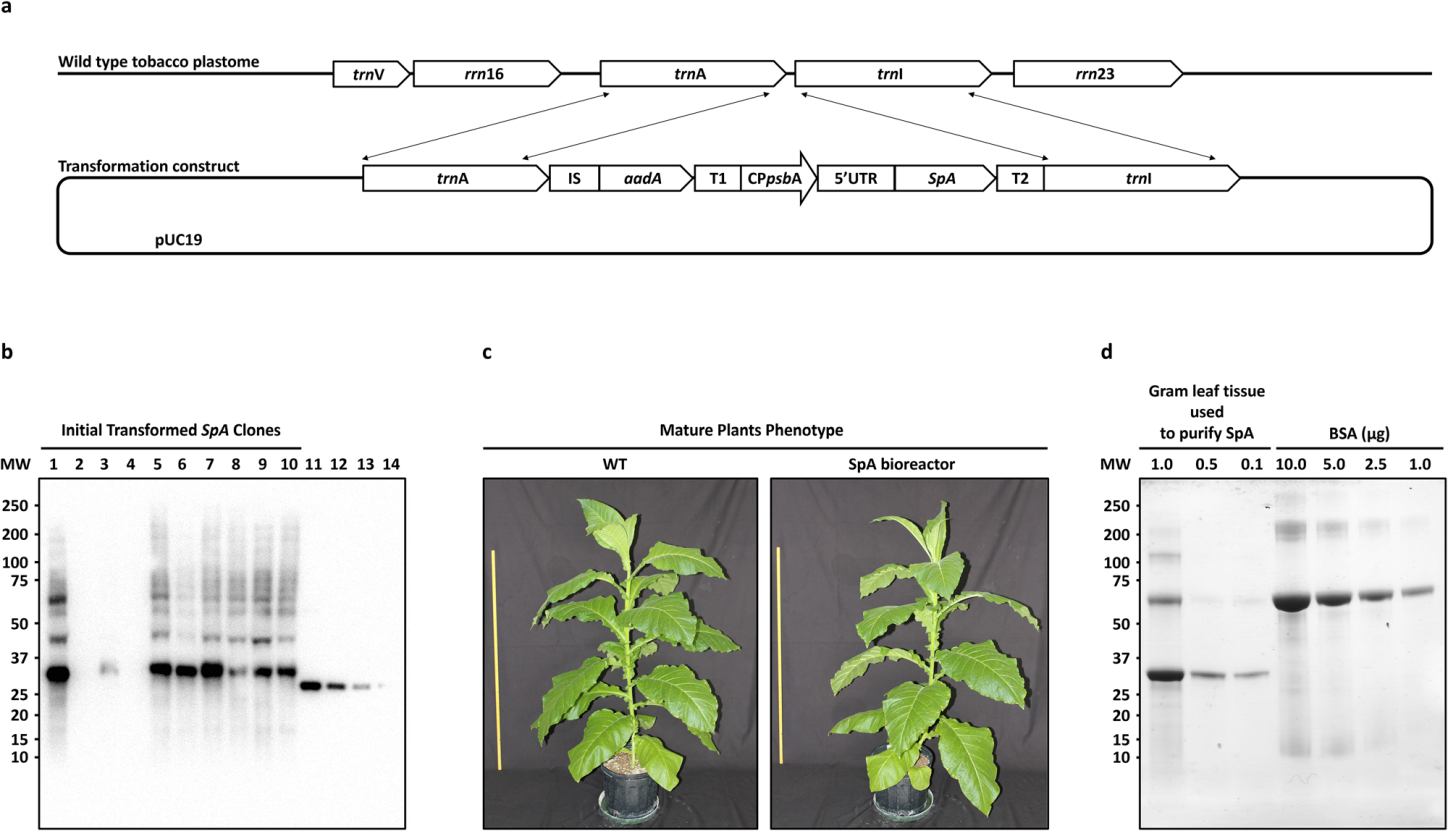
Generation of SpA-expressing bioreactor lines. (**a**) A schematic illustration of the tobacco plastome locus (*trn*A-*trn*I intergenic spacer) targeted for the integration of the *SpA* expression cassette. The transformation construct (pUC19-based) contains *SpA* expression cassette flanked by sequences homologous to *trn*A (left flank) and *trn*I (right flank) of the tobacco plastome (NC_001879), allowing transformation and integration of the cassette into the plastome through homologous recombination (depicted with thin connecting arrows). The *SpA* expression cassette consists of the following elements: “IS”—sequence homologous to the intergenic spacer between *rps*2 and *atp*I genes of tobacco plastome (NC_001879); “aadA”—active selection marker gene encoding aminoglycoside acetyltransferase; “T1”—transcriptional terminator sequence from the *rbc*L gene of the *Populus alba* plastome (NC_008235); “CP *psb*A”—core promoter sequence from the *psb*A gene of tobacco plastome (NC_001879); “5’UTR”—untranslated region of the *psb*A gene of tobacco, or phage T7 gene g10; “*SpA*”—codon-optimized gene sequence encoding the five IgG-binding domains of *S. aureus* protein A fused with a his-tag and a cysteine at the 3’ end; “T2”—transcriptional terminator sequence from the *psb*C gene of the *Populus alba* plastome (NC_008235). (**b**) SDS-PAGE and Western blot-assisted screening of the total soluble protein (TSP) extracts from the initial regenerated clones (lanes 1 through 10, equal amounts of 10 mg extracted leaf tissue per lane) transformed with the synthetic constructs for expression of recombinant SpA, lanes 11 through 14 contain known amounts of a quantifiable control protein (eGFP, 100, 50, 25 and 12.5 ng, respectively); both SpA and eGFP bear the same his-tag probed with anti-his-tag antibody. (**c**) The phenotype of the wild type (WT) and the SpA-expressing bioreactor line plants; a reference 1 m ruler is shown on the left side of the plants. (**d**) SDS-PAGE and stained gel image depicting results of the recombinant SpA purification from various amounts of crude TSP extract (1.0 g, 0.5 g and 0.1 g leaf tissue) using immobilized metal ion affinity chromatography (IMAC). Twenty µL of each of the purified samples were separated on the gel, the yield of the purified SpA was evaluated in each purification sample in comparison with a standard curve of known amounts of Bovine Serum Albumin (BSA, see Methods). Molecular weight (MW) is shown in kilo Daltons.

We used the “toolbox” approach to select, screen and optimize the parameters for the *SpA* gene expression from several synthetic constructs^21^. Clones regenerated from the transformed calli displayed no visible difference from the wild-type untransformed plants when grown in a greenhouse, producing an estimated ~ 250 mg of the recombinant SpA per 1 kg of fresh weight (FW) leaf biomass, whereas ~ 80% of the SpA yield could be purified (Fig. 1). However, we observed significantly lower SpA expression (~ 70–80 mg/kg FW) in the second generation of plants grown from seeds of the initial transformants (data not shown), suggesting that the full line homoplastomy was not achieved before flowering/setting seed. Similar outcome was observed in another bioreactor line produced for expression of an unrelated protein (manuscript in preparation). Currently, the SpA line is being restored to full homoplastomy state by repeated cycles of regeneration on selective media^22,23^.

### Coupling of the recombinant SpA to the resin particles

The recombinant SpA was designed to contain a C-terminal cysteine, a sulfhydryl-containing amino acid, which allowed for spatially-oriented covalent coupling to agarose beads bearing iodoacetyl groups, thus formulating a ligand protein A chromatography resin (PlantA resin, see Methods section). Spatially-oriented coupling was previously shown to be advantageous for achieving improved antibody binding capacity^24^. The plant-produced, purified recombinant SpA was coupled to agarose beads using the SulfoLink Immobilization kit for proteins, the efficacy of the process was monitored by assessing fractions’ protein contents after each step using a spectrophotometer. The concentrated SpA solution containing ~ 2.5 mg of the recombinant protein was initially reduced with addition of 2-mercaptoethylamine-HCl, forming free sulfhydryl groups on the C-terminal cysteines on the recombinant SpA, after which the reduced protein solution was desalted and mixed with the resin agarose beads to achieve the covalent coupling to the iodoacetyl groups present on the resin. By analysing the protein content of the flow-through fractions after the desalting and the coupling stages of the process, we deduced the recovery of 63% and coupling efficiency of 52% (see Methods for a detailed description).

### PlantA resin binding and elution of the monoclonal antibody mAb53

**Figure 2:**
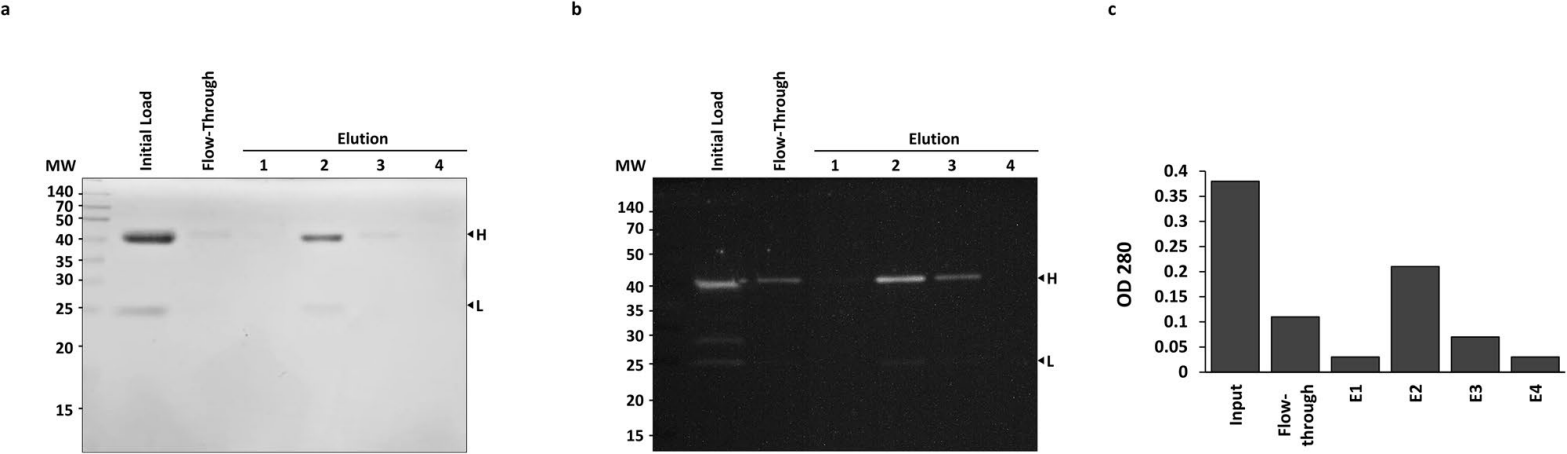
Monoclonal antibody mAb53 binding to and elution from the PlantA resin. Samples from each of the fractions collected in the procedure of binding and elution of mAb53 with PlantA resin were profiled by SDS-PAGE and staining [20 µL samples, (**a**)] and Western blotting with a polyclonal anti-mouse antibody [5 µL samples,** (b**)], as well as analyzed by spectroscopy at 280 nm wavelength (**c**). The samples included initial antibody mAb53 solution loaded on the column (Initial Load), the flow-through fraction (Flow-through), and the four consecutive elutions of the bound antibody mAb53, showing the heavy (H) and the light (L) chains of the IgG molecule. Molecular weight (MW) is shown in kilo Daltons.

We tested the ability of the manufactured PlantA resin to bind and elute IgG-type antibodies with the application of the anti (α) Osteopontin mouse monoclonal antibody 53 (mAb53), which was available in our laboratory at that time. The antibody solution at neutral pH was loaded on the column and briefly incubated on a rocking platform, allowing slight blending of the resin bed. After that, the column was drained of fluid, which was collected as the flow-through fraction. Then, the column was eluted four consecutive times with a resin bed volume of a lower pH buffer and the eluates were collected. All obtained liquid fractions were analysed for the presence of soluble proteins using SDS-PAGE separation with staining, and Western blotting with a polyclonal anti-mouse antibody, as well as tested with spectroscopy. The results presented in Fig. 2 confirmed that mAb53 had bound to and was subsequently eluted from the newly-produced PlantA resin. These results were in accordance with the expected performance of a functional protein A ligand.

*Purification of the monoclonal mouse IgG2a antibodies N86/8 and N86/38 from the supernatants of the producing hybridomas using the chromatography resin PlantA manufactured with plant-sourced protein A ligand.*

**Figure 3:**
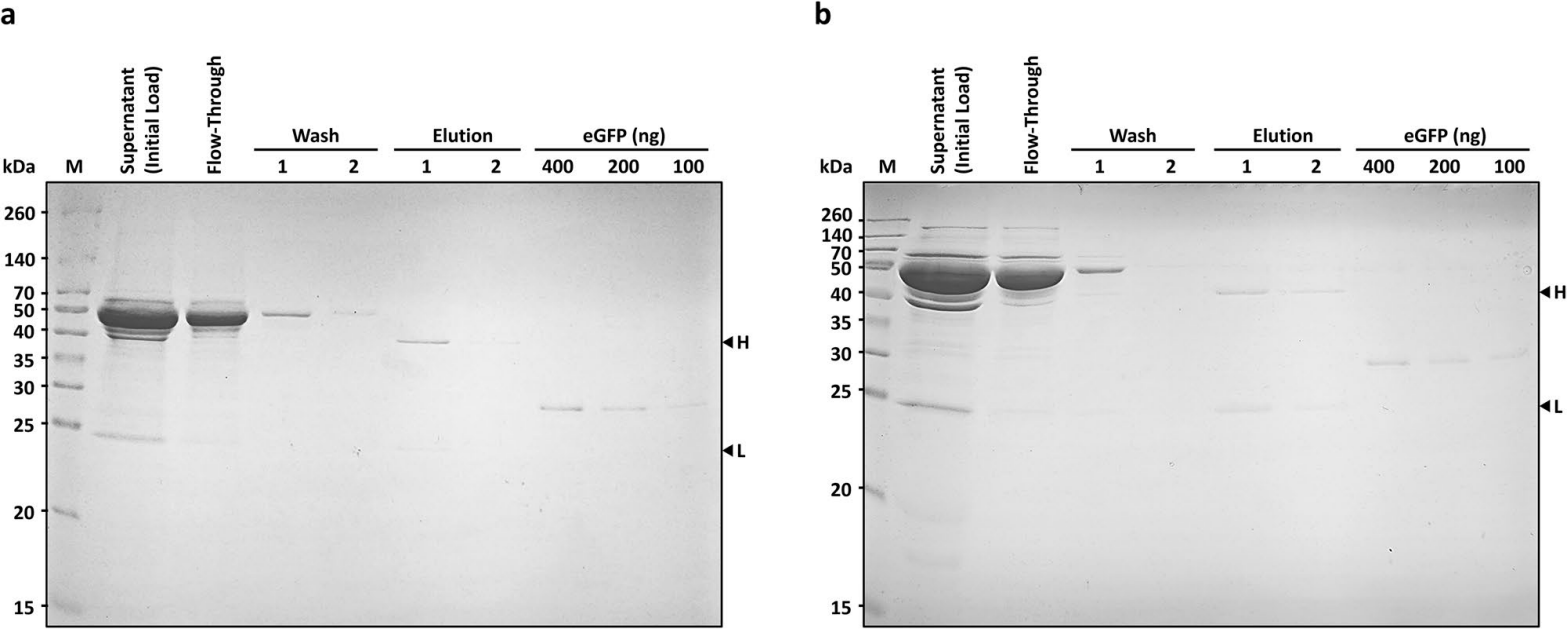
Purification of the monoclonal mouse IgGs from the supernatants of the producing hybridomas N86/8 and N86/38 using the chromatography resin manufactured with the plant-produced protein A ligand (PlantA resin). Fifty µL of each fraction were loaded per well. SDS-PAGE and stained gels results of the initial supernatant (Initial Load) loaded on the column, the flow-through fraction, the two consecutive washes and the two elutions of the purified IgGs, showing the heavy (H) and the light (L) chains of the molecule (indicated by black triangle arrows next to the letters) for (**a**) N86/8 (titer of ~ 31 µg/mL) and (**b**) N86/38 (titer of ~ 47 µg/mL). Standard quantifiable eGFP was present in the amounts of 400, 200, and 100 ng per lane. M—molecular weight marker shown in kilo Daltons (kDa).

We further tested the performance of our PlantA resin in the purification of mAbs from the supernatants of their producing hybridomas. For that, we used supernatants from two hybridoma lines, N86/8 and N86/38, from the Developmental Studies Hybridoma Bank (DSHB, https://dshb.biology.uiowa.edu); those hybridoma lines produce mouse monoclonal antibodies directed against the Green Fluorescent Protein (α GFP) from *Aequorea* (*A*.) *victoria*. Since we have an abundant supply of select recombinant fluorescent proteins (FPs), including a GFP variant, enhanced (e) GFP, all produced from plant bioreactor lines, we opted to purify those specific αGFP mAbs to facilitate their functionality testing by the immunodetection of GFP. These purifications were conducted repeatedly, processing 1 mL of the supernatant per one purification procedure, during which all the liquid fractions were collected, sampled and the protein content was examined by SDS-PAGE separation. Stained SDS-PAGE profile of the obtained fractions (Fig. 2a and b) presented the initial supernatant load, the flow-through fraction, two consecutive washes and two elutions of the antibodies, showing the heavy and the light chains of the molecules in the absence of any visible contamination. The results indicated that the purification procedure was successful, and that the affinity chromatography PlantA resin selectively bound, retained, and eluted the IgGs as designed, eliminating most of the contaminating proteins in the process. Estimations of the efficacy of the manufactured chromatography resin show that 81% and 92% of N86/38 and N86/8 antibody titers, respectively, were successfully purified from the supernatants in one chromatography step (Fig. 3, Table 1).

**Table 1:** Estimated recovery of the mAbs N86/8 and N86/38 after PlantA resin-assisted purification from the supernatants.

mAb	Supernatant titer (µg/mL)	Supernatant volume (mL)	Purified titer (µg/mL)	Purified volume (mL)	% Recovery ± SEM	# Purifications performed (n)
N86/8	31.0	3	7.1	12	92 ± 7.5	3
N86/38	47.0	5	9.5	20	81 ± 16.4	5

### Functional analysis of the purified mAbs N86/8 and N86/38

**Figure 4:**
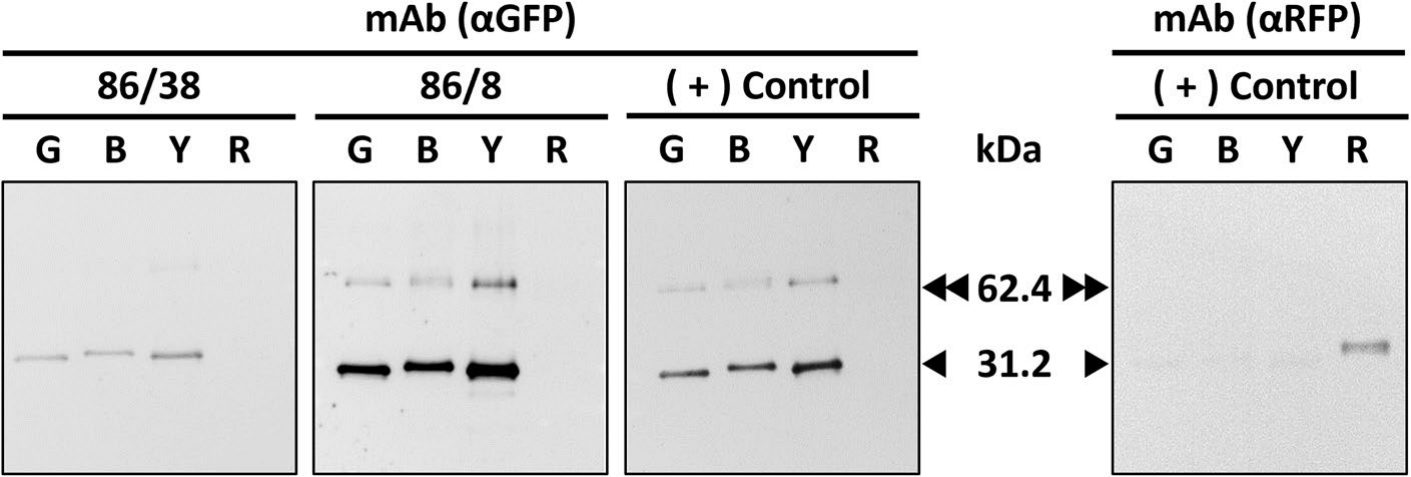
Immunodetection of various recombinant FPs with the purified αGFP mAbs N86/8 and N86/38. Commercially obtained, positive (+) control αGFP and αRFP mAbs and the two purified αGFP mAbs (N86/8 and N86/38) were used as the primary antibody for detection of four different fluorescent proteins by Western blot following SDS-PAGE separation. Four eFPs: Green (G), Blue (B), Yellow (Y) and Red (R) at 100 ng/lane were probed. The molecular weight (kDa) of the GFP monomer (black triangle arrow, 31.2) and dimer (double black triangle arrow, 62.4) are indicated.

We tested the performance of the purified αGFP mAbs N86/8 and N86/38 by applying them as the primary antibodies in the Western blotting-assisted immunodetection of four recombinant enhanced FPs: Green, Blue, Yellow and Red (eGFP, eBFP, eYFP and eRFP), these four proteins were produced from engineered tobacco bioreactor lines and were previously demonstrated to be properly folded and fully functional (sgbiotec.com). The eGFP, eBFP and eYFP are all derivatives of the original GFP protein from *A. victoria* (Uniport P42212) and were expected to react with an αGFP mAb, while the eRFP (Uniport Q9U6Y8) was derived from *Discosoma sp*. and therefore is undetectable by αGFP mAbs, while reactive with a specific αRFP-raised mAb. The expected protein bands of the *A. victoria*-derived FPs of the correct molecular size were produced with immunodetection by the purified mAbs N86/8 and N86/38 as the primary antibodies in Western blot, while the eRFP was not detected. In contrast, eRFP was detected with αRFP mAb used as a control, which further validated the functionality of the PlantA-purified αGFP mAbs^25^. The resulting blots are presented in Fig. 4.

## Discussion

Plants are phototrophic primary producers that use solar energy to convert atmospheric CO_2_ into carbohydrates and create biomass. Knowledge and practice of growing plants allowed humans to develop our civilization as we know it. With the demonstrated ability of plant-based production platforms to address the constraints of cost and scalability, pertinent to the usage of conventional stationary fermenters, harnessing the power of photosynthesis to manufacture various biologics makes perfect sense from economic and environmental perspectives^9,16^.

The global market for therapeutic mAbs is growing fast with expected revenues of $300 billion by 2025^27^. To support the continuation of this growth, as well as expand the affordability of advanced healthcare it is imperative to establish a reliable supply of every component of the production process, striving to employ ecologically friendly and sustainable technologies. Therefore, in this study we assessed the possibility of photosynthesis-driven mass-production of the protein A from *S. aureus*, SpA, which is a standard chromatography ligand used by industry in the process of mAbs purification. To our best knowledge, this is the first report on successful abundant production of a functional variant of SpA in a stable line of transgenic plants, previous work that evaluated transient expression method using agrobacterium‐infiltrated *Nicotiana benthamiana* plants with a transformation construct exploiting nuclear genetic machinery, designed for endoplasmic reticulum retention of the recombinant product, obtained ~ 60 µg of the recombinant SpA per 1 g fresh leaf material sampled six days post-infiltration^28^. We engineered a plant bioreactor line producing a recombinant variant of SpA in chloroplasts; the polypeptide contained all five IgG-binding domains of the native protein fused with a hepta-histidine (his) tag capped with a cysteine residue at the C-terminus^29^. The original plastome-transformed clones yielded ~ 250 µg of the recombinant SpA in crude extract of 1 g of leaf tissue, ~ 200 µg of that purifiable using a simple IMAC procedure. Thus, theoretically extrapolating those observed recombinant SpA yields to biomass generation with the engineered bioreactor line in greenhouse/field settings stipulates production of ~ 1 g of the recombinant SpA ligand from the leaves of ~ 5 mature plants. Given field-grown tobacco plant density of 125,000 plants (up to 250,000!)/hectare with an option to multi-crop twice in the growing season (in southern Ontario), speculatively, up to 25 kg of the recombinant SpA can be extracted from 1 hectare seasonal crop, with a potential to producing ~ 1000 L of an affinity chromatography resin^30,31^. The seeds generated by the engineered bioreactor line displayed strict cytoplasmic heredity of the SpA expression trait, when germinated on selective medium. However, when transferred to soil and grown to maturity, those second generation plants displayed a much lower SpA yield in mature stage, suggesting that the original bioreactor line was heteroplastomic^23^. Currently the line is being stabilized to its homoplastomic state using repeated cycles of tissue culture regeneration on selective medium^32^.

The C-terminal hepta-histidine tag allowed efficient and inexpencive IMAC-based purification of the soluble recombinant SpA protein ligand from crude leaf tissue extracts; the C-terminal cysteine enabled covalent, spatially-oriented immobilization of the SpA molecule onto the beaded agarose support granules, thus rendering the hepta-his-tag a linker between the granules’ surfaces and the SpA ligand. We estimated that ~ 1 mg of the ligand was covalently linked to the beads in 1 mL of 50% slurry in our coupling reaction, making it ~ 1.7 × 10^16^ molecules of the recombinant SpA ligand. Based on that, we reasoned that the number of mAb molecules that can potentially bind to, and be retained by the ligand molecules on the support granules would be ~ 6.8 × 10^16^, which translates into ~ 16 mg of an mAb, considering the bivalent binding model for the ligand SpA-IgG interaction^33,34^. The manufactured chromatography resin with the photosynthetically-sourced ligand protein A (PlantA resin) was functionally tested in binding and elution of mAb53, which revealed that ~ 80% of the initial 730 µg of the mAb53 applied to the PlantA resin column (2 mL of 50% slurry) were retained and subsequently eluted (Fig. 2). At present it is difficult to explain why binding/elution at 100% rates haven’t been achieved, given the theoretical binding capacity of the manufactured PlantA resin. Further quantitative experiments and analyses are needed to establish the optimal ratio of the ligand amounts present on the resin support granules for achieving maximized performance of the affinity chromatography resin PlantA.

We set up our experiments to purify the mAbs 86/38 and 86/8 from hybridoma supernatants quantitatively so that we could visually present all fractions of the procedure separated by SDS-PAGE in one 10-well stained gel. Processing 1 mL of a supernatant at a time allowed efficient, repeatable, and consistent purification of up to 92% of the mAbs’ initial titers. The purified mAbs were fully functional in specific immunodetection of their antigen GFP and its derivatives, validating the practicality of the PlantA resin application.

In conclusion, in this scientific report we describe formulation of PlantA—a chromatography affinity resin harboring recombinant ligand protein A from *S. aureus* sourced from an engineered photosynthetic plant bioreactor. Repeated purifications of mAbs of the IgG-type from the supernatants of the producing hybridoma lines using the PlantA resin were performed successfully. Future experiments will focus on optimization of the ligand density of the resin, as well as exploring new protein affinity ligands as candidates for photosynthetic mass-production.

## Methods

### Bioreactor line engineering

Tobacco cv. 81V9 plastome was engineered to express and accumulate a recombinant SpA variant comprizing the five IgG-binding domains (E, D, A, B and C)^4^ from *S. aureus* protein A (UniProt identifier P02976), fused with a hepta-histidine tag and with a cystein constituting the C-terminal amino acid of the peptide with the estimated molecular mass of ~ 35.4 kDa. Transplastomic plants expressing the recombinant SpA were generated with the biolistic technique, using 0.6 micron gold particles^30,33^. The detailed description of the expression cassette used and its plastome integration locus is found in the legend to Fig. 1a and in the patent application No. PCT/CA2023/050,008. The engineered bioreactor line was provided by Solar Grants Biotechnology Inc. (SGB Inc., sgbiotec.com). All permissions for including the plant material/samples were obtained from SGB Inc. All experimental research work in this study, including the collection of plant material, comply with relevant institutional, national, and international guidelines and legislation.

### Purification of recombinant SpA from leaf biomass

Recombinant SpA was extracted from leaves in a soluble form by milling the frozen leaf tissue with mortar and pestle in liquid nitrogen and adding phosphate buffered saline (PBS, pH = 7.4, 1:3 w/v), supplemented with 10 mM leupeptin and 2 mM PMSF (Extraction buffer). The extract was cleared by centrifugation at 13,000Xg for 20 min, filtered through Whatman Grade 1 Filter Paper (Cytiva, Cat. # 1,001,320) and purified using immobilized metal affinity chromatography (IMAC) Sepharose His SpinTrap column (Cytiva, Cat. # 28,932,171) following the manufacturer’s protocol. For quantification of the amount of the purifiable recombinant SpA (Fig. 1d) 3 g of fresh leaf tissue were extracted in 15 mL (1/5 w/v) and then different volumes of the extract (5 mL, 2.5 mL and 0.5 mL) were loaded on separate IMAC columns, representing 1 g, 0.5 g and 0.1 g leaf tissue extracted. The columns were further washed and, subsequently, the purified recombinant SpA was eluted in the final volume of 700 µL of Elution Buffer (PBS, pH = 7.4 supplemented with 500 mM imidazole).

### Coupling of plant-produced recombinant SpA to agarose beads

To couple protein A to agarose beads, the Sulfolink Immunomobilization kit for proteins (ThermoFisher #44,995) was used according to the manufacturer’s instructions. Briefly, 450 µL of partially purified protein A (6.9 mg/mL) was added to 500 uL of sample preparation buffer (0.1 M sodium phosphate, 5 mM EDTA-Na; pH 6.0). An aliquot (10 µL) was retained for analysis by SDS-PAGE. The rest of the diluted protein A was added to a vial containing 6 mg of 2-mercaptoethylamine•HCl (final concentration 50 mM) and incubated at 37 °C for 90 min to reduce any disulfide bonds into free sulfhydryl groups. Next, the sample was desalted on the Zeba™ desalt spin column provided with the kit. The flow-through (1.5 mL) was analyzed using a Nanodrop for protein content (1.3 mg/mL; recovery 63%). Then, 1 mL of coupling buffer (50 mM Tris, 5 mM EDTA-Na; pH 8.5) was added to the reduced protein A sample. An aliquot (10 µL) was retained for analysis by SDS-PAGE and the rest of the sample was added to the column containing the Sulfolink resin, mixed by rocking for 15 min at room temperature, followed by incubation for 30 min without mixing. The column was centrifuged at 1000 g for 1 min to obtain the flow-through (2.5 mL). The protein content of the flow-through was 0.37 mg/mL (coupling efficiency 52.2%). An aliquot of the flow-through (10 uL) was retained for analysis by SDS-PAGE. The column was washed three times with 2 mL wash solution (1.0 M NaCl, 0.05% NaN_3_), followed by a wash with 2 mL of coupling buffer. Finally, non-specific binding sites were blocked by the addition of 2 mL coupling buffer containing 50 mM cysteine to the column and incubation for 15 min at room temperature on a rocking platform followed by incubation for 30 min without mixing. The column was washed with PBS and stored in degassed PBS containing 0.05% NaN_3_ at 4 °C until use.

### Binding and elution of mAb53 using the manufactured chromatography PlantA resin with photosynthetically-sourced protein A

The prepared affinity resin with photosynthetically-sourced protein A ligand (PlantA resin, 2 mL of a 50% slurry) was equilibrated at room temperature with 6 mL of PBS, pH = 7.4, by flow-through. Next, 730 µg of the monoclonal antibody mAb53 (Enzo Life Sciences, Cat. # ADI-905–629-100) in 2 mL PBS was allowed to enter the column bed by gravity. Subsequently, 0.2 mL PBS was added, and the binding reaction was allowed to proceed for 15 min at room temperature on a rocking platform. Next, the column was centrifuged at 1000 × *g* for 1 min and the clarified unbound material was collected. Then, 1 mL PBS was added to the column followed by centrifugation at 1000 × *g* for 1 min. Thereafter, the column was washed 4 times with 2 mL of PBS with repeated centrifugations at 1000 × *g* for 1 min. Finally, the monoclonal antibody was eluted using 8 mL 0.1 M glycine (pH = 3.0). Four fractions of 2 mL were collected in tubes already containing 100 µL of 1.5 M Tris–HCl (pH = 8.7), Fig. 1.

### Purification of the mAbs N86/8 and N86/38 using the manufactured chromatography PlantA resin with photosynthetically-sourced protein A

Hybridomas N86/8 and N86/38 produce mouse monoclonal IgG2a antibodies raised against Green Fluorescent Protein (GFP) from *A. victoria.* Supernatants from hybridomas N86/8 and N86/38, containing mAb titers of ~ 31 µg/mL and ~ 47 µg/mL, respectively, were obtained from the Developmental Studies Hybridoma Bank (DSHB, https://dshb.biology.uiowa.edu).

In the antibody purification process 1 mL of supernatant was mixed with 1 mL PBS, pH = 7.4, and then loaded on the PlantA resin column (1 mL of a 50% slurry) pre-equilibrated with 6 mL PBS, and then incubated 15 min at room temperature on a rocking platform for binding, followed by two subsequent washes with PBS (pH = 7.4, 4 mL each) and two elutions of the purified mAbs (2 mL each; 0.1 M glycine, pH = 3.0, collected by gravity flow-through into tubes already containing 100 µL of 1.5 M Tris–HCl, pH = 8.7), Fig. 2. In between the purification procedures the PlantA resin column was stored at 4 °C with addition of 2 mL PBS, pH = 7.4, supplemented with 0.02% sodium azide.

### Western blotting

Western blots for immunodetection of the recombinant proteins in this study were carried out as described previously^25^. Screening of the initially generated transplastomic clones for recombinant SpA expression utilized separation of crude leaf tissue extracts by 12% sodium dodecyl sulfate (SDS)-polyacrylamide gel electrophoresis (PAGE) and the primary anti-his-tag mouse antibody (Cytiva, Cat. # 27–4710-01).

Four FPs (eGFP, eBFP, eYFP and eRFP, 100 ng each) were separated by SDS-PAGE and transferred onto a polyvinylidene difluoride membrane (Thermo Fisher Scientific, Cat. # 88,585), with subsequent blocking of the membrane using skimmed milk. The purified mAbs N86/8 and N86/38 were used as the primary antibodies in the procedure, at concentrations of 0.5 µg/mL, as recommended by DHSB. Polyclonal goat anti-mouse secondary antibodies were obtained from Invitrogen, Cat. # W10808, and used at a working dilution of 1:500, according to manufacturer’s instructions.

### Statistical analysis

The generated images of the SDS-PAGE-stained gels contained the datasets representing the repeated purifications of mAbs from the hybridoma supernatants (Fig. 3; Supplementary Information). Densitometry of the protein bands was performed using the Image J software (https://imagej.nih.gov/ij/index.html), generating the numerical data. The statistical analysis was based on the linear regression of the data obtained from the calibration points 100, 200 and 400 ng of the control protein (eGFP) in each data set. The goodness-of-fit for linear regression models was ascertained by the calculated R-squared factor (R^2^), which was very close to the unity in all cases (0.97 ≤ R^2^ ≤ 0.99), validating the linear models. The linear model in each dataset was used to calculate the amount of the heavy and the light chains of the IgG molecule in the gel, extrapolating on the measurements of the control eGFP.

### Supplementary Information

Supplementary Information 1.

Supplementary Information 2.

## Data Availability

All data generated and analyzed during this study are included in this published article and its Supplementary Information (Supplementary Dataset File). Enquiries regarding availability of the generated plant bioreactor line for SpA expression and accumulation should be sent to igor.k@sgbiotec.com.

## Abbreviations

mAbs: Monoclonal antibodies
IgG: Immunoglobulin type gamma
GFP: Green Fluorescent protein
BFP: Blue Fluorescent protein
YFP: Yellow Fluorescent protein
RFP: Red Fluorescent protein
PBS: Phosphate-buffered saline
PMSF: Phenylmethylsulfonyl fluoride
IMAC: Immobilized metal ion affinity chromatography
SpA: *Staphylococcus (S.) aureus* Protein A

## References

[1] Yang O, Qadan M, Ierapetritou M (2020). Economic analysis of batch and continuous biopharmaceutical antibody production: A review. J. Pharm. Innov..

[2] Hou Y (2022). The application of hollow fiber cartridge in biomedicine. Pharmaceutics.

[3] Hjelm H, Hjelm K, Sjijquist J (1972). Protein A from *Staphylococcus aureus*. Its isolation by affinity chromatography and its use as an immunosorbent for isolation of immunoglobulins. FEBS Lett..

[4] Moks T (1986). Staphylococcal protein A consists of five IgG-binding domains. Eur. J. Biochem..

[5] Rigi G, Ghaedmohammadi S, Ahmadian G (2019). A comprehensive review on staphylococcal protein A (SpA): Its production and applications. Biotechnol. Appl. Biochem..

[6] Nian R (2016). Advance chromatin extraction improves capture performance of protein A affinity chromatography. J. Chromatogr. A.

[7] Kelly B (2009). Industrialization of MAb production technology. MAbs.

[8] Li Z, Carstensen B, Rinas U (2014). Smart sustainable bottle (SSB) system for *E. coli* based recombinant protein production. Microb. Cell Fact..

[9] Xu S, Gavin J, Jiang R, Chen H (2017). Bioreactor productivity and media cost comparison for different intensified cell culture processes. Biotechnol. Prog..

[10] Hao J, Xu L, He H, Du X, Jia L (2013). High-level expression of Staphylococcal Protein A in *Pichia pastoris* and purification and characterization of the recombinant protein. Protein Expr. Purif..

[11] Buyel JF, Twyman RM, Fischer R (2015). Extraction and downstream processing of plant-derived recombinant proteins. Biotechnol. Adv..

[12] Huebbers JW, Buyel JF (2021). On the verge of the market—plant factories for the automated and standardized production of biopharmaceuticals. Biotechnol. Adv..

[13] McNulty MJ (2020). Techno-economic analysis of a plant-based platform for manufacturing antimicrobial proteins for food safety. Biotechnol. Progress..

[14] Shanmugaraj B, Bulaon CJI, Phoolcharoen W (2020). Plant molecular farming: A viable platform for recombinant biopharmaceutical production. Plants.

[15] Buyel JF, Twyman RM, Fischer R (2017). Very-large-scale production of antibodies in plants: The biologization of manufacturing. Biotechnol. Adv..

[16] Ridgley LA (2023). Killer to cure: Expression and production costs calculation of tobacco plant-made cancer-immune checkpoint inhibitors. Plant Biotechnol. J..

[17] Liu YX (2023). Advancing approach and toolbox in optimization of chloroplast genetic transformation technology. J. Integr. Agric..

[18] An Y, Wang Y, Wang X, Xiao J (2022). Development of chloroplast transformation and gene expression regulation technology in land plants. Front. Plant Sci..

[19] Ruf S, Karcher D, Bock R (2007). Determining the transgene containment level provided by chloroplast transformation. Proc. Natl. Acad. Sci. USA..

[20] Maliga, P. Editor. *Chloroplast biotechnology methods and protocols methods in molecular Biology 1132*. http://www.springer.com/series/7651.

[21] Kolotilin I (2022). Plant-produced recombinant cytokines IL-37b and IL-38 modulate inflammatory response from stimulated human PBMCs. Sci. Rep..

[22] Sriraman P, Silhavy D, Maliga P (1998). Transcription from heterologous rRNA operon promoters in chloroplasts reveals requirement for specific activating factors. Plant Physiol..

[23] Lutz KA, Maliga P (2008). Plastid genomes in a regenerating tobacco shoot derive from a small number of copies selected through a stochastic process. Plant J..

[24] Zhang X, Duan Y, Zeng X (2017). Improved performance of recombinant protein A immobilized on agarose beads by site-specific conjugation. ACS Omega.

[25] Gallagher S, Winston SE, Fuller SA, Hurrell JGR (2008). Immunoblotting and immunodetection. Curr. Protoc. Mol. Biol..

[26] Buyel JF (2019). Plant molecular farming–integration and exploitation of side streams to achieve sustainable biomanufacturing. Front. Plant Sci..

[27] Lu RM (2020). Development of therapeutic antibodies for the treatment of diseases. J. Biomed. Sci..

[28] Kurppa K, Reuter LJ, Ritala A, Linder MB, Joensuu JJ (2018). In-solution antibody harvesting with a plant-produced hydrophobin—protein A fusion. Plant Biotechnol. J..

[29] Kanje, S., Scheffel, J., Nilvebrant, J. & Hober, S. Engineering of Protein A for improved purification of antibodies and Fc-fused proteins. in *Approaches to the Purification, Analysis and Characterization of Antibody-Based Therapeutics* 35–54 (Elsevier, 2020). doi:10.1016/B978-0-08-103019-6.00002-3.

[30] Reynolds B, McGarvey B, Todd J (2022). Agronomics of high density tobacco (*Nicotiana tabacum*) production for protein and chemicals in Canada. Biocatal. Agric. Biotechnol..

[31] Yang XH, Huan LM, Chu XS, Sun Y, Shi QH (2018). A comparative investigation of random and oriented immobilization of protein A ligands on the binding of immunoglobulin G. Biochem. Eng. J..

[32] Svab Z, Hajdukiewicz P, Maliga P (1990). Stable transformation of plastids in higher plants. Proc. Natl. Acad. Sci. USA..

[33] Nelson JT (2015). Mechanism of immobilized protein A binding to immunoglobulin G on nanosensor array surfaces. Anal. Chem..

[34] Sjöquist J, Meloun B, Hjelm H (1972). Protein A isolated from staphylococcus aureus after digestion with lysostaphin. Eur. J. Biochem..

[35] Svab Z, Maliga P (1993). High-frequency plastid transformation in tobacco by selection for a chimeric aadA gene. Proc. Natl. Acad. Sci. USA..

