# Supplementary material for: Chromatography affinity resin with photosynthetically-sourced protein A ligand
Source: Sci Rep. 2024 Apr 15;14:8714. doi: 10.1038/s41598-024-59266-2 (PMC11018848; doi:10.1038/s41598-024-59266-2)
Supplement: Supplementary file 1 — Supplementary Information 1. [file 41598_2024_59266_MOESM1_ESM.pdf]

ImageJ Data

Purification	Band	ng protein/band		
N86/38 - I	E1-Hc		1	4532.4
	E1-Lc		2	2977.6
~47 µg/mL	E2-Hc		3	1617.4
	E2-Lc		4	1624.8
	eGFP	400	5	2901.5
	eGFP	200	6	1520.3
	eGFP	100	7	815.1

N86/8 - I	E1-Hc		1	3396.6
	E1-Lc		2	479.3
~31 µg/mL	E2-Hc		3	418.2
	E2-Lc		4	149.4
	eGFP	400	5	2833.9
	eGFP	200	6	1433.0
	eGFP	100	7	513.8

N86/38 - II	E1-Hc		1	3123.2
	E1-Lc		2	1408.3
	E2-Hc		3	2267.9
	E2-Lc		4	1132.9
	eGFP	400	5	3589.9
	eGFP	200	6	1486.7
	eGFP	100	7	597.0

N86/8 - II	E1-Hc		1	2908.9
	E1-Lc		2	1082.3
	E2-Hc		3	462.7
	E2-Lc		4	193.8
	eGFP	400	5	2445.8
	eGFP	200	6	1504.9
	eGFP	100	7	897.4

**N86/38 - III**	**E1-Hc**		**1**	**5072.5**
	**E1-Lc**		**2**	**2094.8**
	**E2-Hc**		**3**	**1976.8**
	**E2-Lc**		**4**	**448.2**
	**eGFP**	**400**	**5**	**3152.2**
	**eGFP**	**200**	**6**	**1714.3**
	**eGFP**	**100**	**7**	**438.7**

**N86/8 - III**	**E1-Hc**		**1**	**1876.4**
	**E1-Lc**		**2**	**1093.9**
	**E2-Hc**		**3**	**543.3**
	**E2-Lc**		**4**	**195.0**
	**eGFP**	**400**	**5**	**1982.0**
	**eGFP**	**200**	**6**	**1006.4**
	**eGFP**	**100**	**7**	**268.5**

N86/38 - IV	E1-Hc		1	1941.9
	E1-Lc		2	4916.8
	E2-Hc		3	336.0
	E2-Lc		4	985.8
	eGFP	400	5	3936.5
	eGFP	200	6	2178.7
	eGFP	100	7	1509.4

N86/38 - V	E1-Hc		1	2177.4
	E1-Lc		2	2803.9
	E2-Hc		3	346.2
	E2-Lc		4	422.5
	eGFP	400	5	4540.4
	eGFP	200	6	2773.5
	eGFP	100	7	2060.8

Gels Images

N86/38 - I

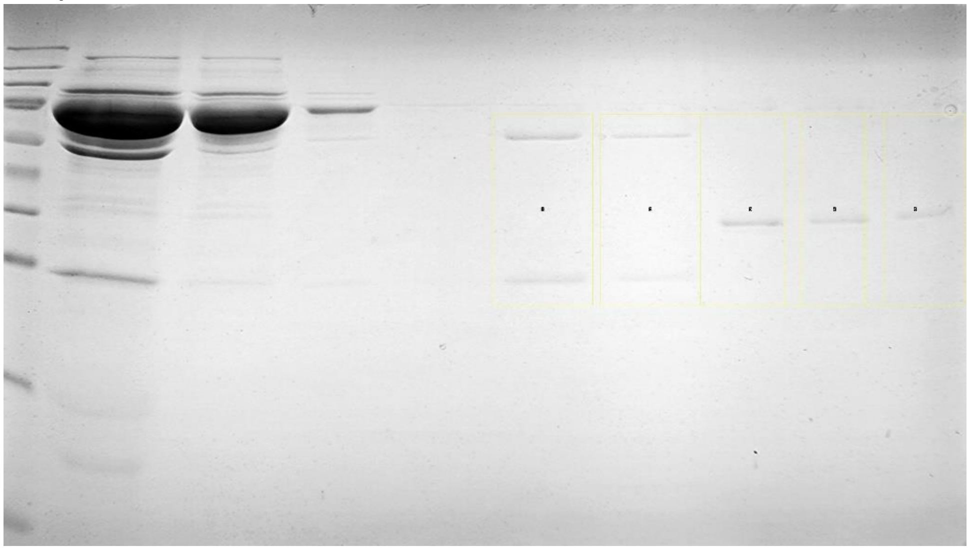

N86/8 - I

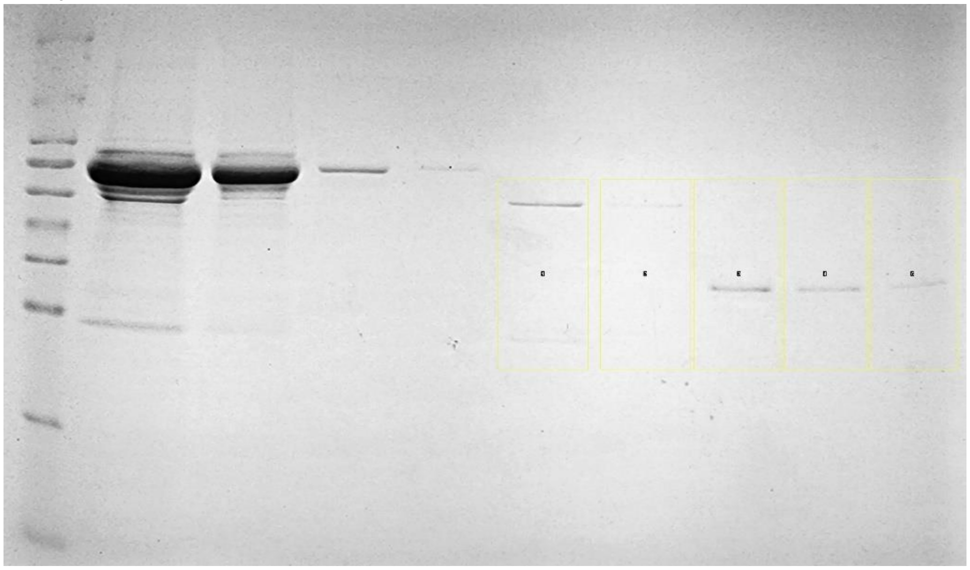

N86/38 - II

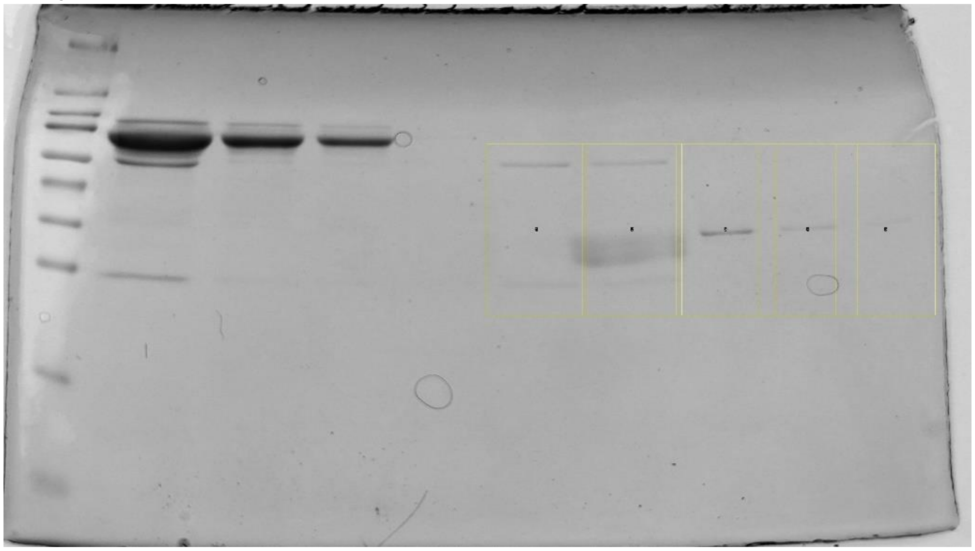

N86/8 - II

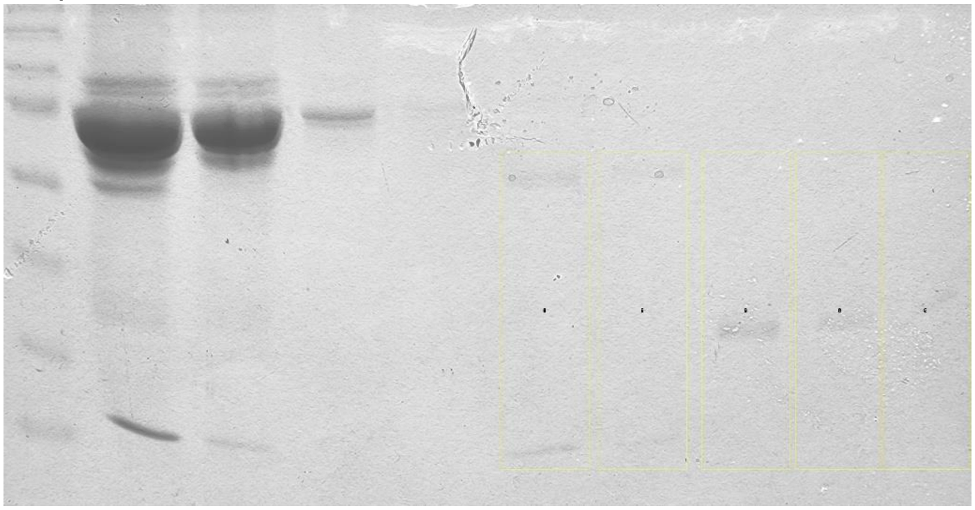

N86/38 - III

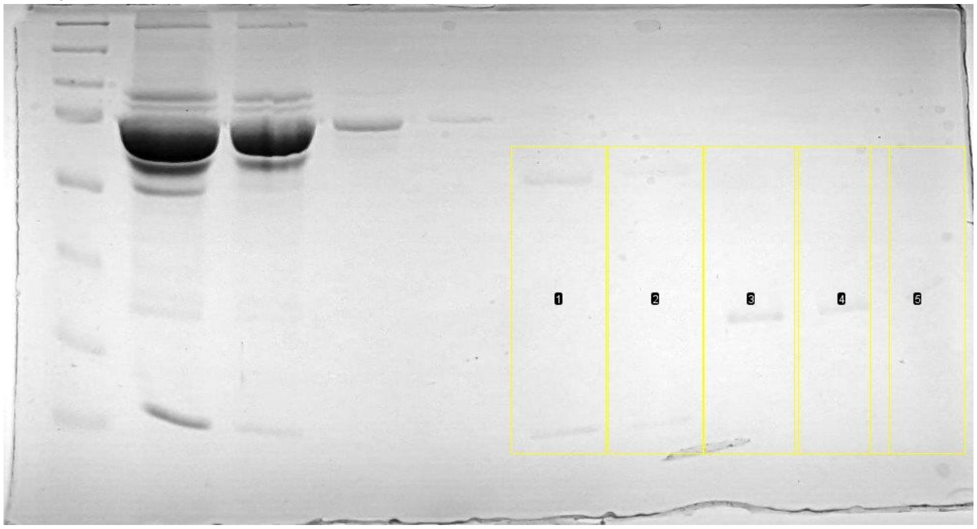

N86/8 - III

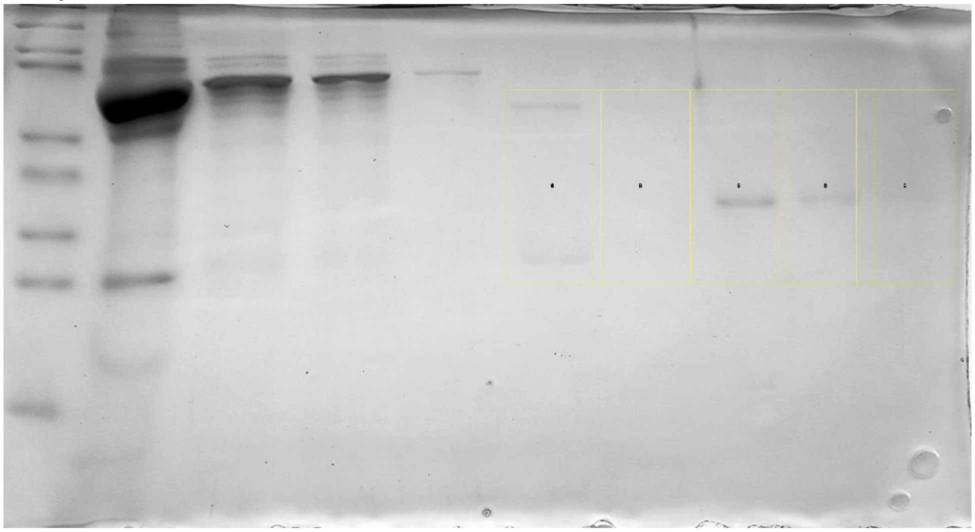

N86/38 - IV

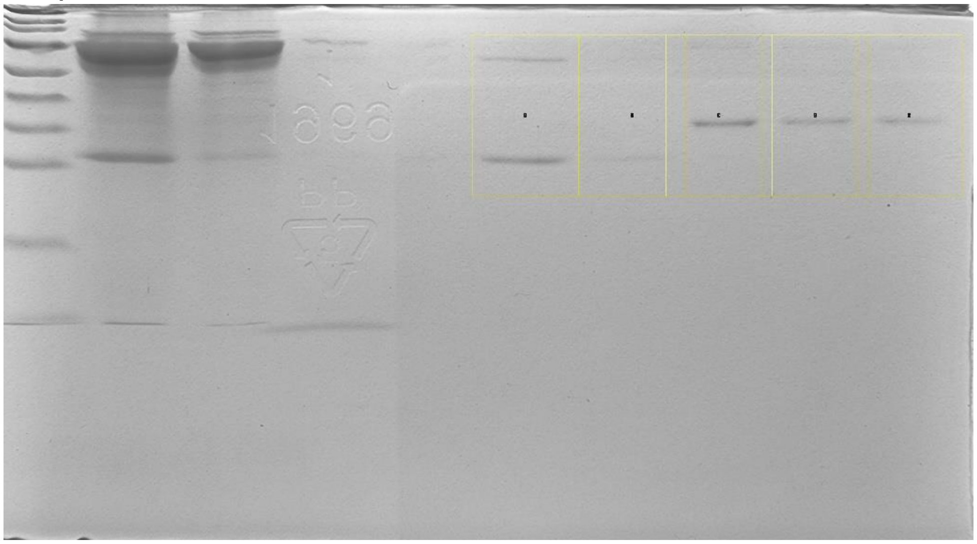

N86/38 - V

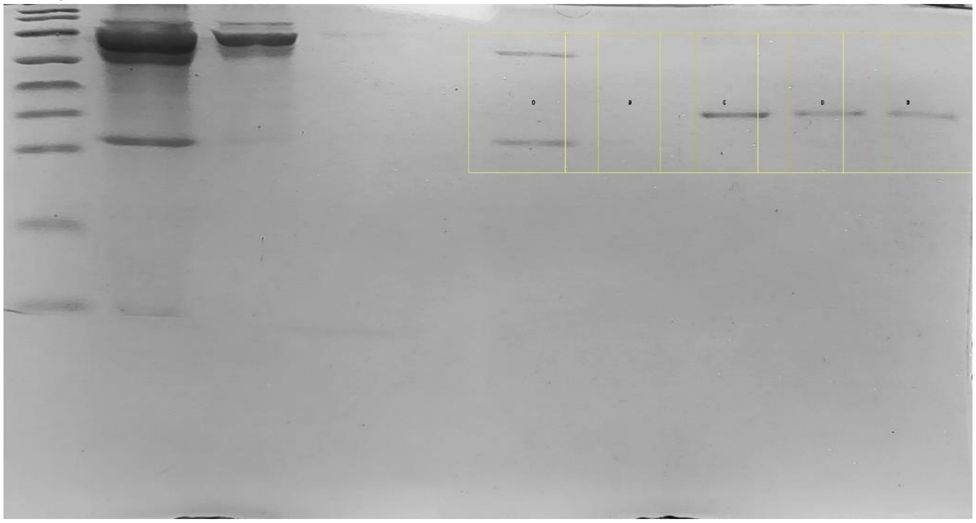

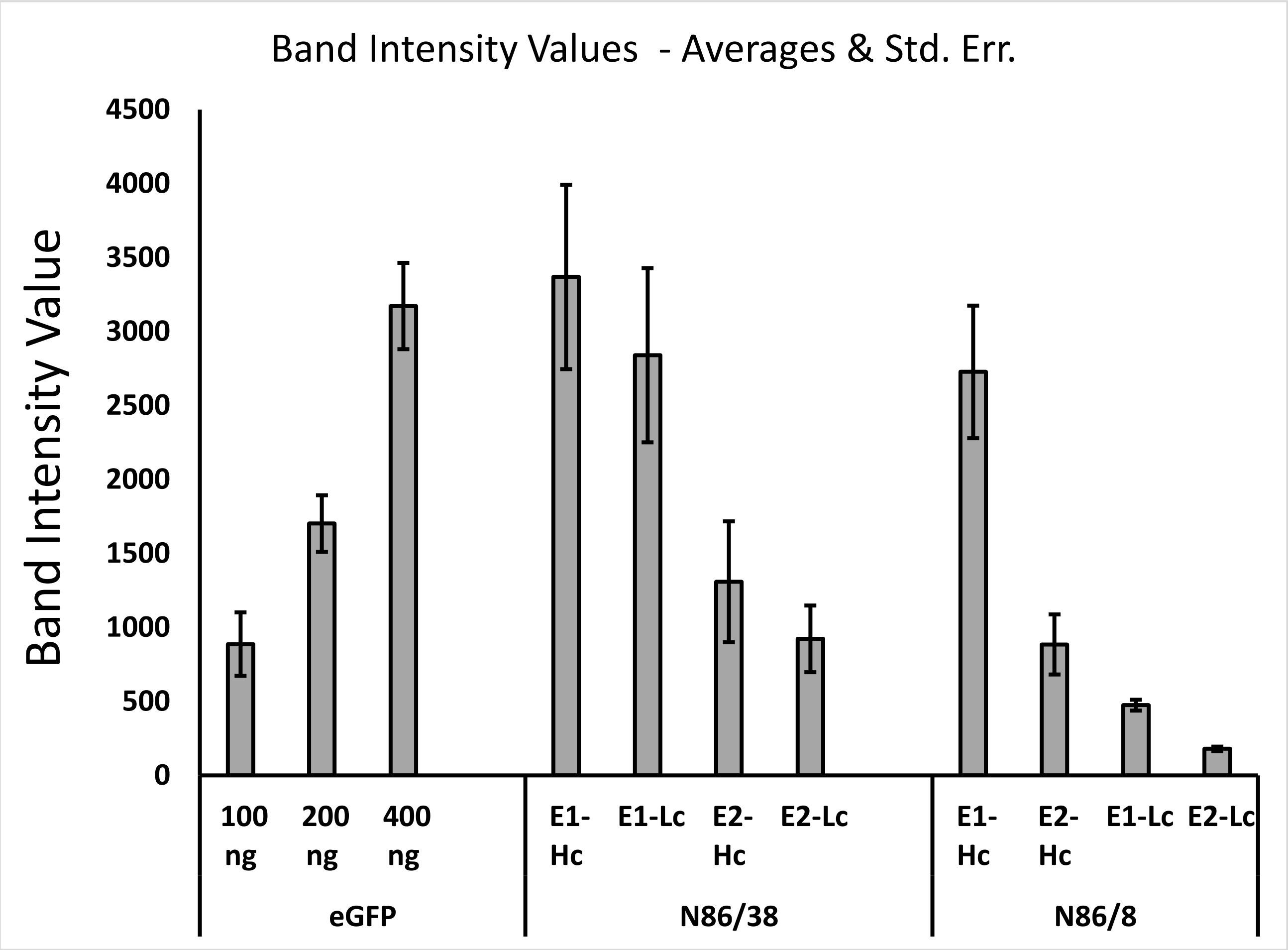

Averages for bands intensity values & STD ERR

		Band intensity value	STD ERROR	Average mAb Recovery (ng)	Per mL
eGFP	100 ng	887.6	214.5		
	200 ng	1702.2	191.3		
	400 ng	3172.7	291.6		
N86/38	E1-Hc	3369.5	623.4	190428	9521
	E1-Lc	2840.3	589.0		
	E2-Hc	1308.9	408.3		
	E2-Lc	922.8	225.4		
N86/8	E1-Hc	2727.3	448.1	85212	7101
	E2-Hc	885.1	203.1		
	E1-Lc	474.7	36.7		
	E2-Lc	179.4	15.0		

Individual purifications regression graphs

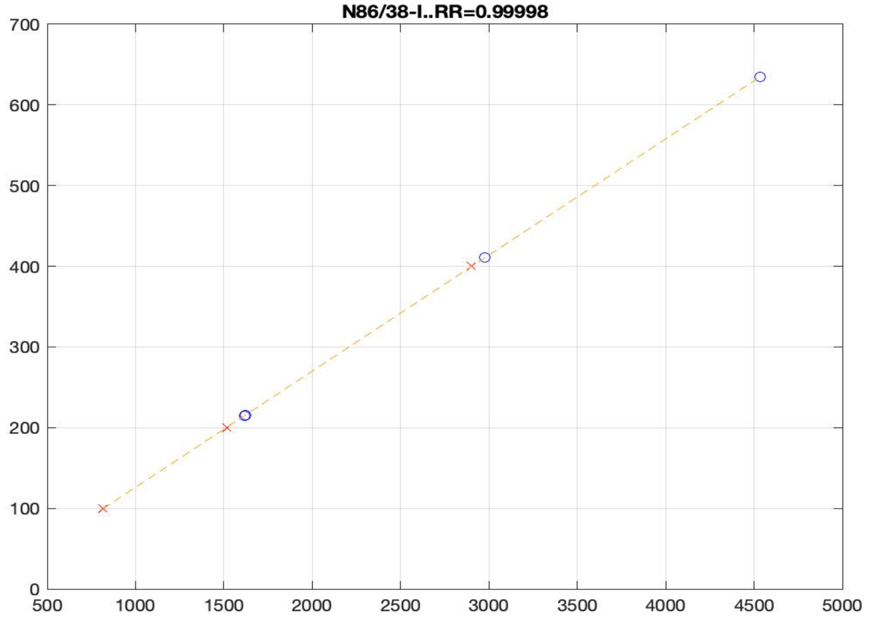

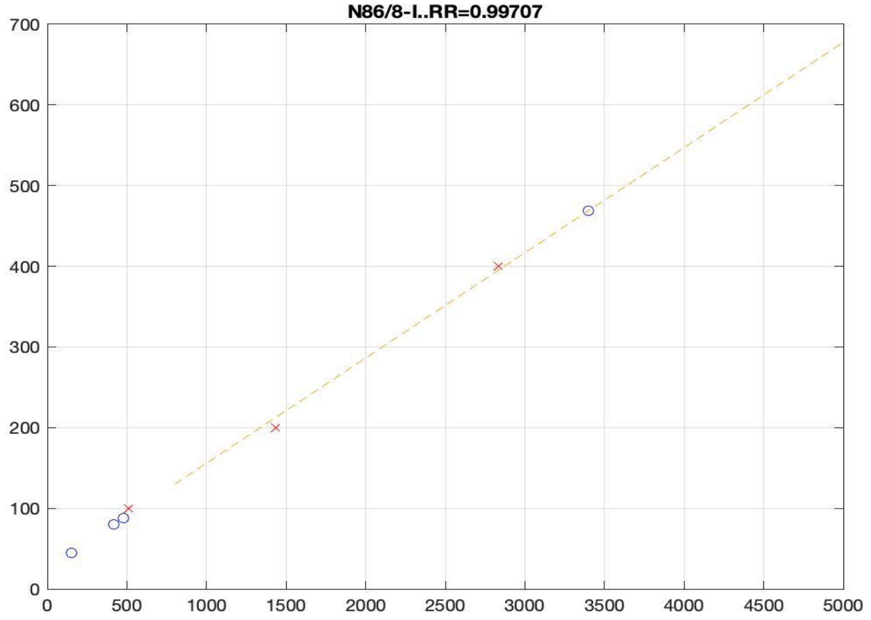

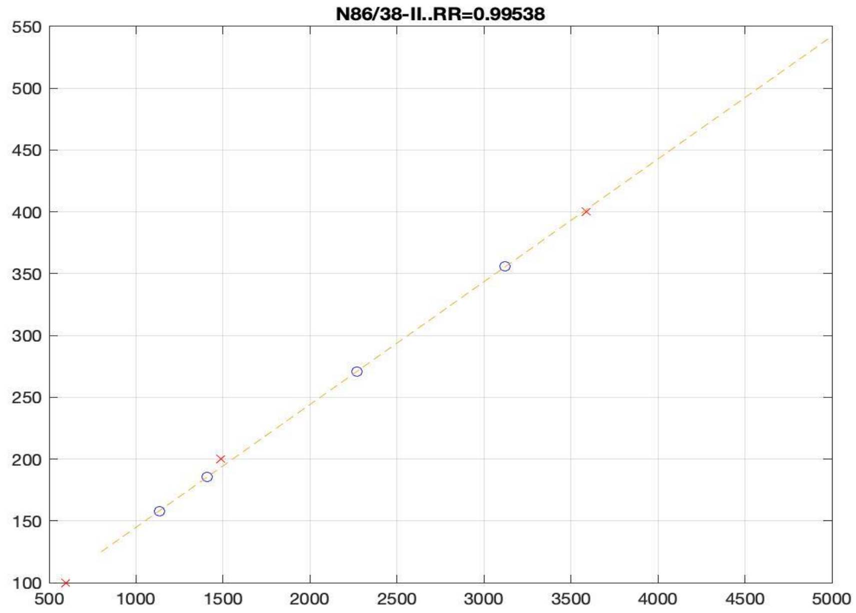
N86/38-II..RR=0.99538
550
500
450
400
350
300
250
200
150
100
500
1000
1500
2000
2500
3000
3500
4000
4500
5000

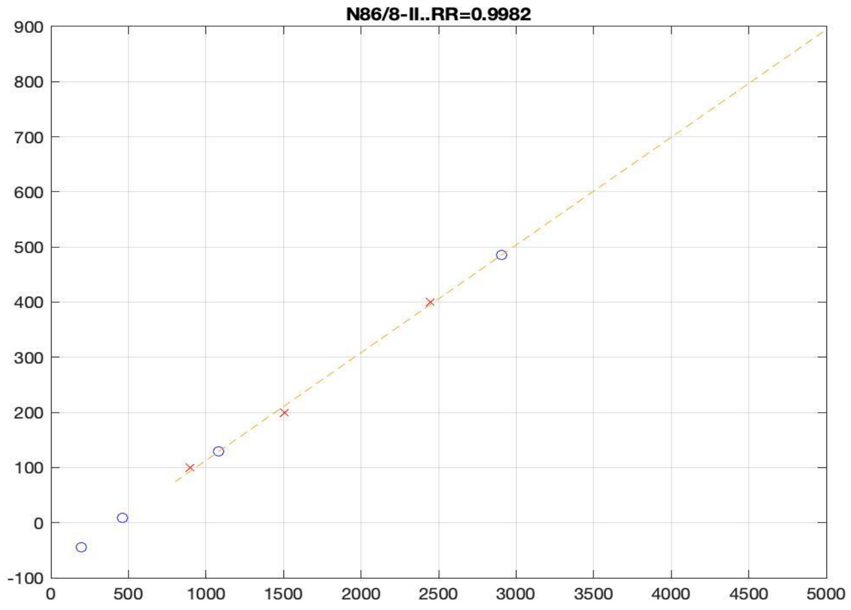
N86/8-II..RR=0.9982
900
800
700
600
500
400
300
200
100
0
-100
0
500
1000
1500
2000
2500
3000
3500
4000
4500
5000

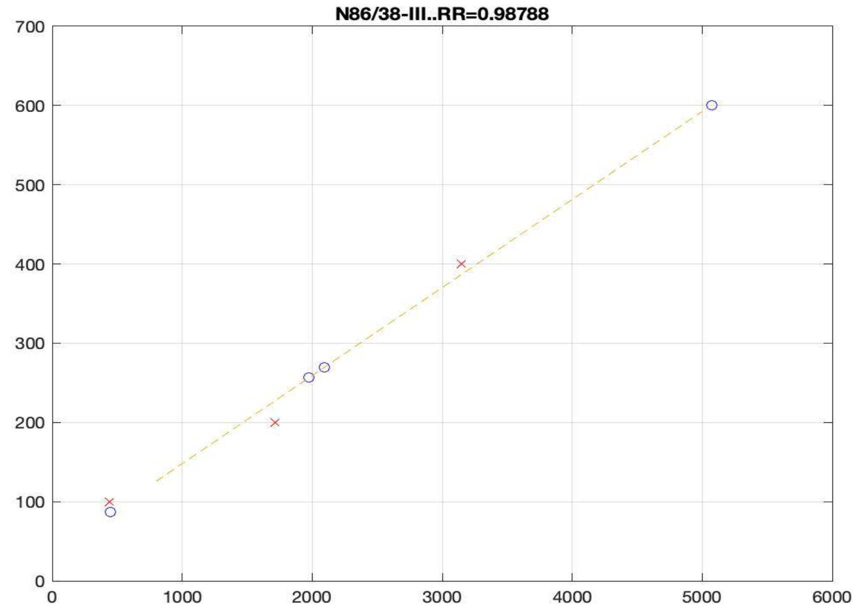
N86/38-III..RR=0.98788
700
600
500
400
300
200
100
0
0
1000
2000
3000
4000
5000
6000

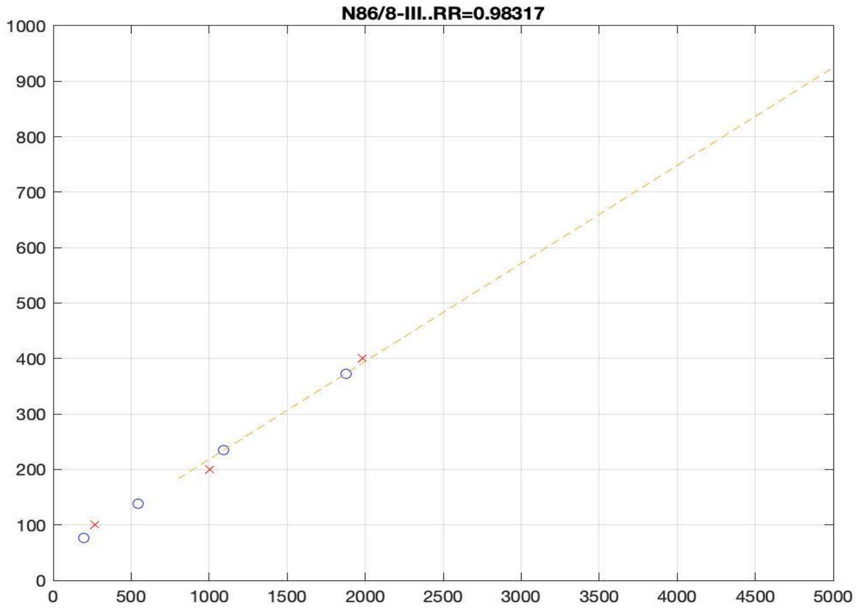
N86/8-III..RR=0.98317
1000
900
800
700
600
500
400
300
200
100
0
0
500
1000
1500
2000
2500
3000
3500
4000
4500
5000

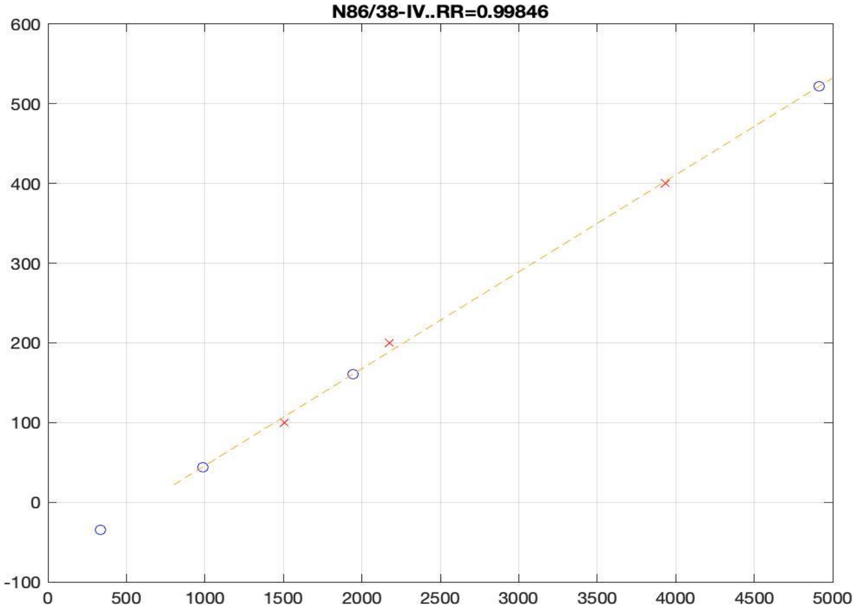
N86/38-IV..RR=0.99846
600
500
400
300
200
100
0
-100
0
500
1000
1500
2000
2500
3000
3500
4000
4500
5000

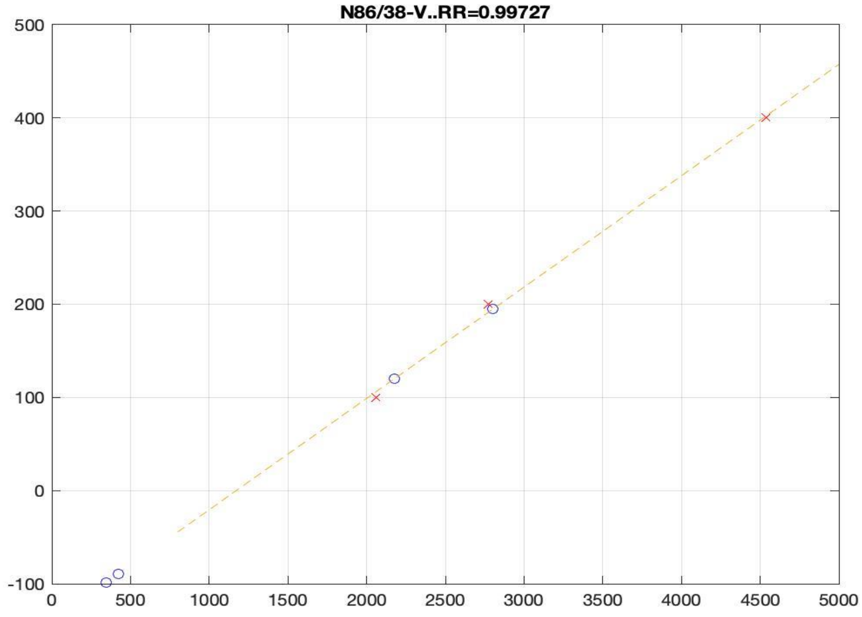
N86/38-V..RR=0.99727
500
400
300
200
100
0
-100
0
500
1000
1500
2000
2500
3000
3500
4000
4500
5000

Calculations of the % of mAbs recovery

N86/38-I
R^2=0.99998

Protein	Calib/Pred (ng) in 50 µL	Measured	Total E
eGFP	100.0	815.1	
eGFP	200.0	1520.3	
eGFP	400.0	2901.0	

E1-Hc	634.6	4532.4	25382.2
E1-Lc	410.7	2977.6	16428.7
E2-Hc	214.9	1617.4	8595.8
E2-Lc	216.0	1624.8	8638.5

N86/8-I
R^2=0.99707

	Calib/Pred (ng)	Measured	Total E
eGFP	100.0	513.8	
eGFP	200.0	1433.0	
eGFP	400.0	2833.9	

E1-Hc	468.5	3396.6	18739.0
E1-Lc	88.0	479.0	3519.1
E2-Hc	80.0	418.0	3200.9
E2-Lc	45.0	149.4	1799.8

N86/38-II
R^2=0.99638

	Calib/Pred (ng)	Measured	Total E
eGFP	100.0	597.0	
eGFP	200.0	1486.7	
eGFP	400.0	3589.9	

E1-Hc	355.7	3123.2	14227.4
E1-Lc	185.4	1408.3	7415.0
E2-Hc	270.7	2267.9	10829.8
E2-Lc	158.0	1132.9	6321.0

N86/8-II
R^2=0.9982

	Calib/Pred (ng)	Measured	Total E
eGFP	100.0	897.4	
eGFP	200.0	1504.9	
eGFP	400.0	2445.8	

E1-Hc	485.9	2908.9	19436.6
E1-Lc	129.1	1082.3	5162.4
E2-Hc	8.0	462.7	320.5
E2-Lc	4.5	193.8	180.0

N86/38-III
R^2= 0.98788

	Calib/Pred (ng)	Measured	Total E
eGFP	100.0	438.7	
eGFP	200.0	1714.3	
eGFP	400.0	3152.2	

E1-Hc	600.6	5072.5	24024.8
E1-Lc	269.6	2094.8	10784.6
E2-Hc	256.5	1976.8	10260.0
E2-Lc	86.6	448.2	3463.2

N86/8-III
R^2= 0.98317

	Calib/Pred (ng)	Measured	Total E
eGFP	100.0	268.5	
eGFP	200.0	1006.4	
eGFP	400.0	1982.0	

E1-Hc	373.0	1876.4	14920.8
E1-Lc	234.8	1093.9	9391.8
E2-Hc	137.5	543.3	5501.3
E2-Lc	76.0	195.0	3040.3

N86/38-IV
R^2=0.99846

	Calib/Pred (ng)	Measured	Total E
eGFP	100.0	1509.4	
eGFP	200.0	2178.7	
eGFP	400.0	3936.5	

E1-Hc	160.4	1941.9	6416.5
E1-Lc	522.2	4916.8	20887.6
E2-Hc	22.3	336.0	890.4
E2-Lc	44.1	985.8	1765.6

N86/38-V
R^2= 0.99727

	Calib/Pred (ng)	Measured	Total E
eGFP	100.0	2060.8	
eGFP	200.0	2773.0	
eGFP	400.0	4540.4	

E1-Hc	120.1	2177.4	4805.3
E1-Lc	195.0	2803.9	7799.8
E2-Hc	16.8	346.2	672.0
E2-Lc	20.5	422.5	820.1

N86/38-I Total mAb in supernatant (ng)	N86/38-I Grand Total mAb purified (ng)	% Recovery
47000	59045	125.6

N86/8-I Total mAb in supernatant (ng)	N86/8-I Grand Total mAb purified (ng)	% Recovery
31000	27259	87.9

N86/38-II	N86/38-II	
Total mAb in supernatant (ng)	Grand Total mAb purified (ng)	% Recovery
47000	38793	82.5

N86/8-II	N86/8-II	
Total mAb in supernatant (ng)	Grand Total mAb purified (ng)	% Recovery
31000	25100	81.0

N86/38-III	N86/38-III	
Total mAb in supernatant (ng)	Grand Total mAb purified (ng)	% Recovery
47000	48533	103.3

N86/8-III	N86/8-III	
Total mAb in supernatant (ng)	Grand Total mAb purified (ng)	% Recovery
31000	32854	106.0

N86/38-IV	N86/38-IV	
Total mAb in supernatant (ng)	Grand Total mAb purified (ng)	% Recovery
47000	29960	63.7

N86/38-V	N86/38-V	
Total mAb in supernatant (ng)	Grand Total mAb purified (ng)	% Recovery
47000	14097	30.0

Average N86/8 % recovery	91.6
Average N86/38 % recovery	81.0

Final:

ng mAb in 5 mL sup: N86/38	235000
ng mAb in 3 mL sup: N86/8	85212
