# Supplementary material for: Chromatography affinity resin with photosynthetically-sourced protein A ligand
Source: Sci Rep. 2024 Apr 15;14:8714. doi: 10.1038/s41598-024-59266-2 (PMC11018848; doi:10.1038/s41598-024-59266-2)

Original Blots Used to Compile Figure 1:

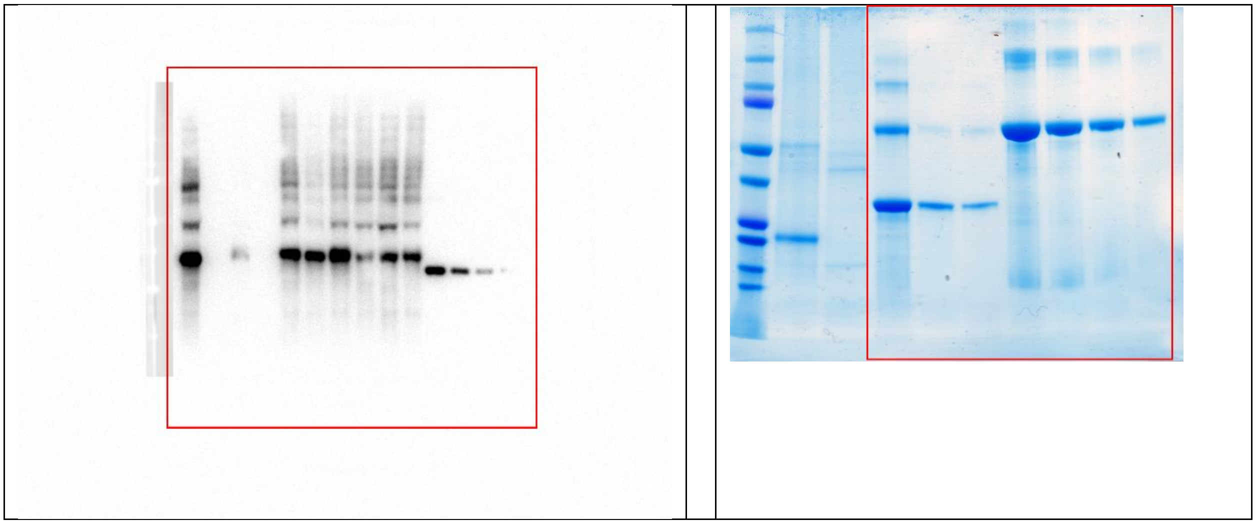

Completed Figure 1:

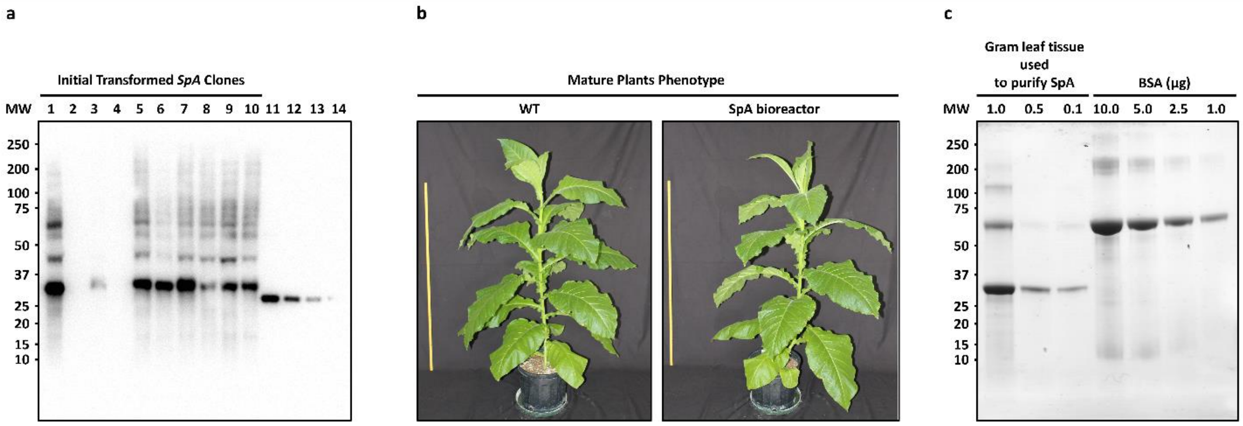

Original Blots Used to Compile Figure 2:

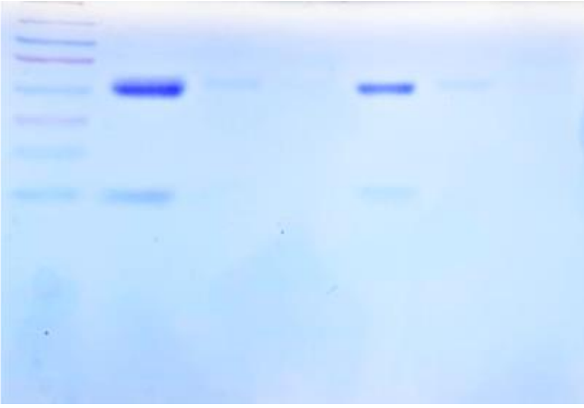

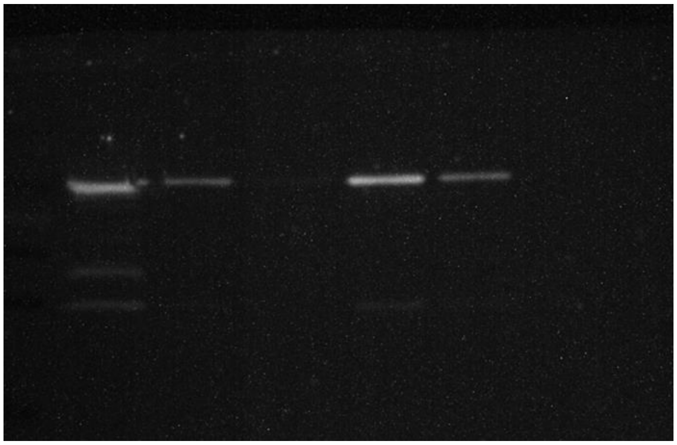

Completed Figure 2:

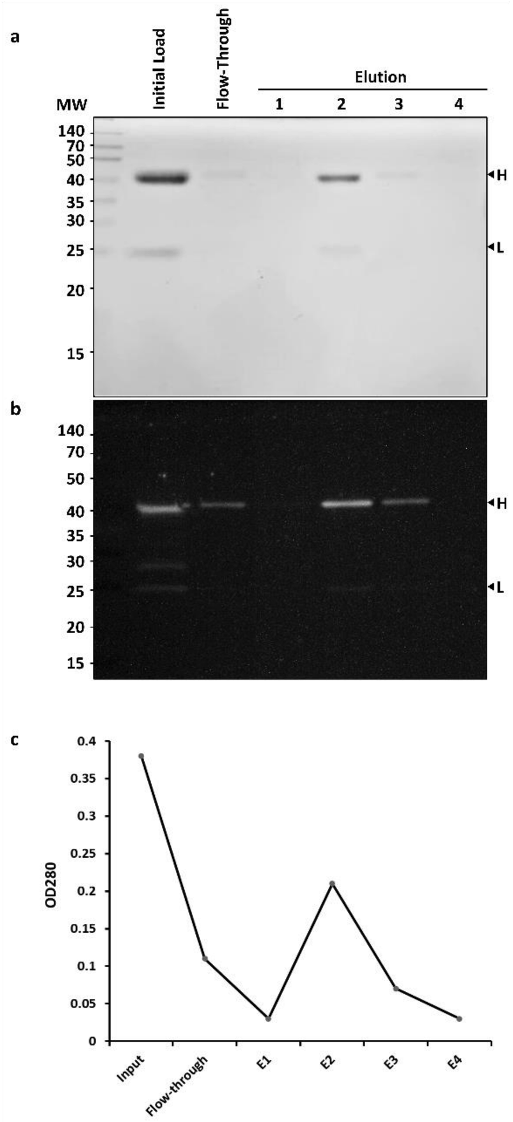

Original Blots Used to Compile Figure 3:

Purification of N86/38 antibody

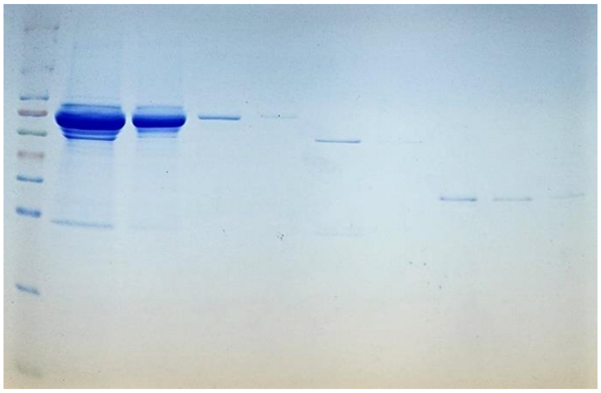

Purification of N86/8 antibody

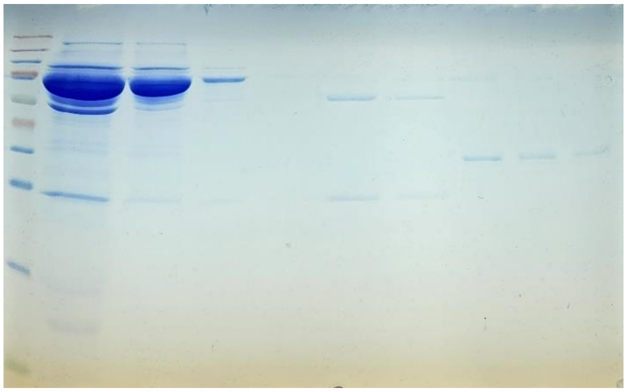

Completed Figure 3:

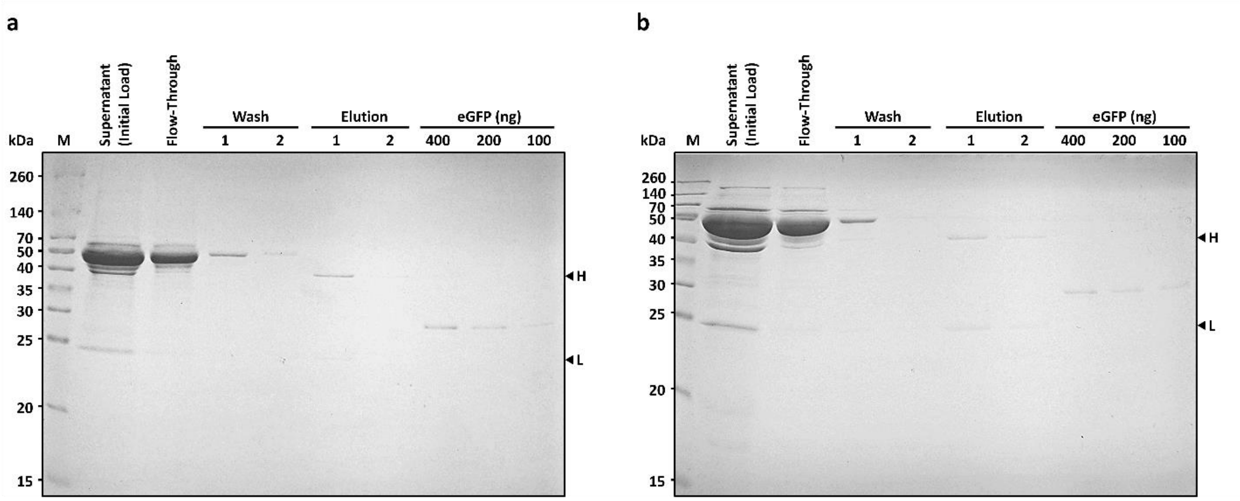

Original Blots Used to Compile Figure 4:

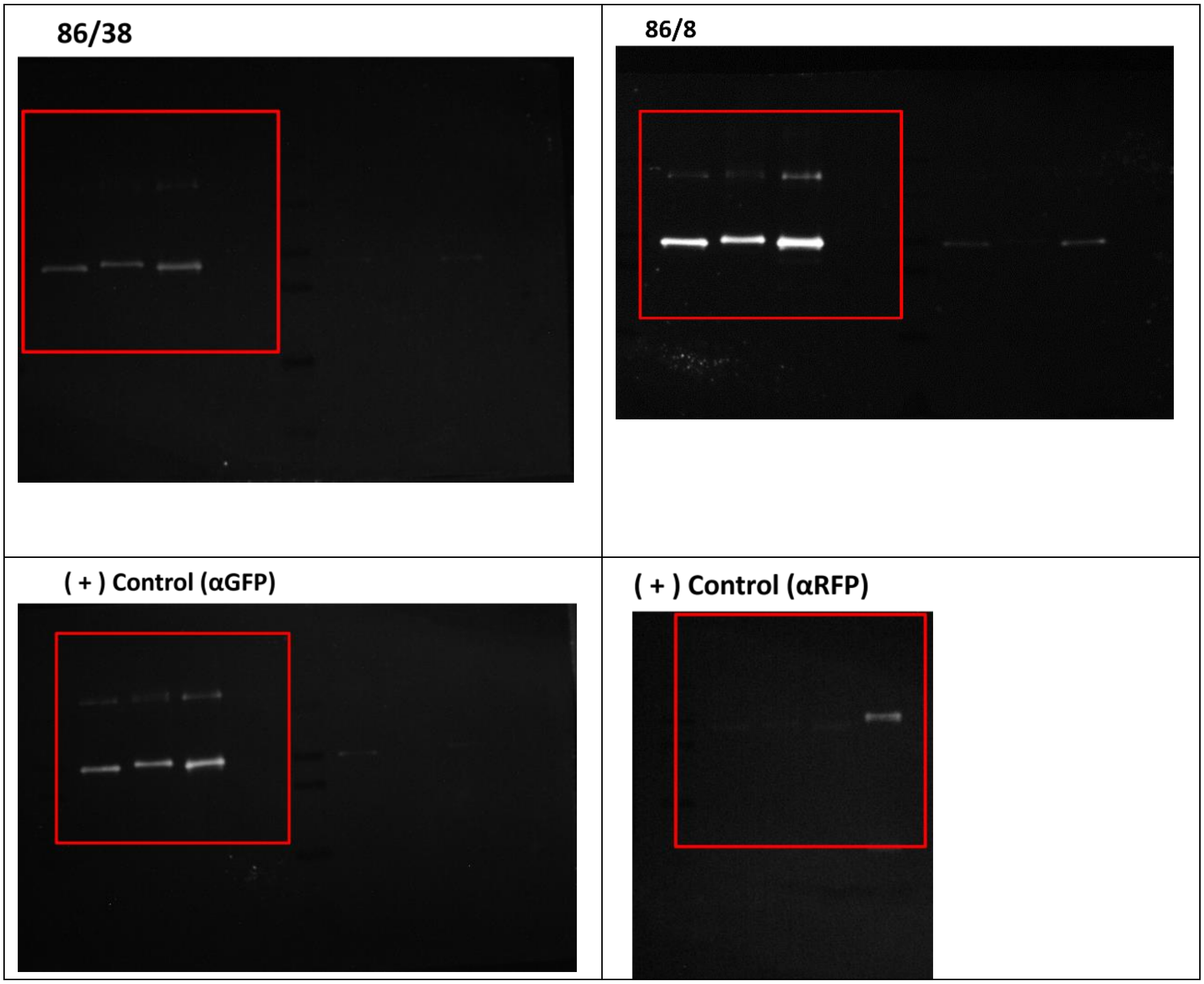

Completed Figure 4:

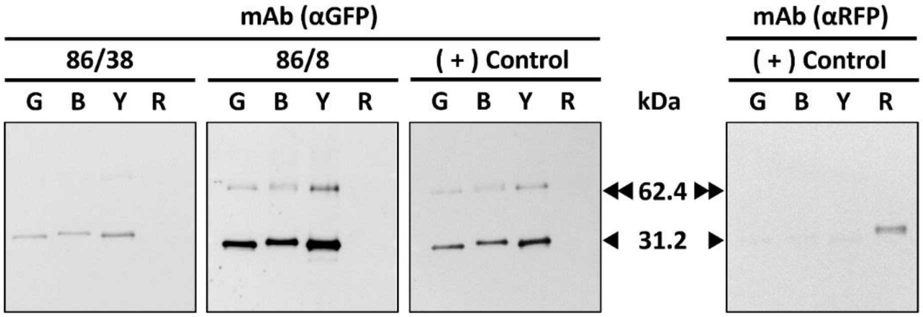

Supplement: Supplementary file 2 — Supplementary Information 2. [file 41598_2024_59266_MOESM2_ESM.pdf]
